# Chronic Constipation

**Published:** 1896-10

**Authors:** Jno. W. Kinney

**Affiliations:** San Antonio


For the Texas Medical Journal.

### CHRONIC CONSTIPATION.

BY JNO. W. KINNEY, M. D., SAN ANTONIO.

[Read at West Texas Medical Society.]

WHEN I was requested by our esteemed President to open the discussion on "Chronic Constipation," my first thought was that he had given me a very dry subject. Since then, however, my mind has undergone a radical change. To-night, I view my subject with almost the zeal of a recent convert, and look upon it as a very comprehensive one; one that should and does interest the physician and surgeon, from a scientific standpoint, and the great mass of humanity from a sense of personal discomfort which it has at one time or another placed at their door.

As I am only to open the discussion on this subject, my remarks, for the sake of brevity, shall be devoted almost exclusively to the causation and treatment of this troublesome affection—principally the treatment, for the cause is simple in a majority of cases.

Chronic constipation may be due to a great many causes. Mechanical obstruction, either congenital or acquired, feeble peristalsis, scanty intestinal secretions, debility, starvation, ingestion of food that is most all assimilated, sedentary habits, and the failure, on the part of the patient, to heed the calls of nature, all tend to produce a slothful state of the large intestine.

Mechanical obstructions, which are usually found in the rectum and near the anus, are, as a rule, due to fibrous bands or excessive folds of the mucous membrane. Piles sometimes act as mechanical obstructions. Fissure, fistula, pruritis and ulceration of the anus may also act as mechanical obstructions. The pain which the act of defecation brings about when any of the above conditions exist, causes an involuntary constriction of the sphincter ani, which virtually amounts to a mechanical obstruction.

Chronic constipation is usually considered, and in the vast majority of cases is, a functional disorder. Feeble peristalsis and debility may be the result of disease, and engraft the habit upon any individual. Starvation and the ingestion of highly nutritious food may result in chronic constipation by their failure to furnish sufficient refuse matter to stimulate the muscular tunic of the lower bowel to action. Scanty intestinal secretions cause a slothful state of the large bowel by failing to sufficiently liquify the fecal matter.

Inattention on the part of the patient to answer the calls of nature, is by far the most important cause of intestinal inertia. Business engagements, false, yet at the same time essential, modesty, and lack of a suitable place to perform the act, often causes us to postpone defecation. By this postponement we allow the gut to become overdistended, thus impairing the contractibility of its muscular fibres and blunting the sensibility of its nerves. In course of time this becomes a chronic condition and calls for our attention on account of the inconvenience which it causes, and its importaoce as a factor in the causation of other diseases.

The treatment of chronic constipation, when due to mechanical obstruction, is either palliative or curative. About the only palliative treatment worthy of mention is the enemata—this will usually bring away the fecal mass. Purgatives should never be resorted to in these cases when the obstruction is purely mechanical, for obvious reasons. To cure one of these cases, the cause must be removed, which can only be done by resorting to surgical measures.

Chronic constipation, as a functional disorder, must be cured "quickly, safely and pleasantly." It requires time, patience and perseverance on the part of both physician and patient.

The prophylactic treatment in this day of preventive medicine should first claim our attention. The people should be educated to that point where they can realize that it is just as important, if not more so, to answer the calls of the system, when it desires to dispose of its refuse matter, as it is to answer the calls for more food to be assimilated.

Our schools should be provided with competent teachers of physiology and its allied branches, that the young might have the importance of the above dictum impressed upon their minds. School children, at the present time, are often forced to remain in the school room and busy their minds trying to prevent the involuntary evacuation of fæces, which, at every comfortable moment, they plead in vain with their teacher to permit them to be excused. There should be at least one privy for every twenty-five pupils, while in this city and in many others there are only about one to every one hundred. These privies should be kept clean and comfortable that one might not dread to enter them, or be in a hurry to leave when circumstances compel them to go there. Parents and teachers should ever impress upon those under their care the importance of keeping the emunctories open and to have a regular time for the performance of defecation. This is a habit easily acquired and conducive of more good than most any other.

The curative treatment of chronic constipation is complex and varied in the extreme. The remedies recommended as infallible are so numerous that they argue their own uselessness. Only a few of the practical ones shall receive mention at my hands. Usually it is best to commence the treatment of intestinal inertia by an enema which softens and removes the hardened masses which have accumulated in the rectum and sigmoid flexure. No possible harm can result from the use of the enema if proper attention be given to the anatomy of the parts while using them. The most important step toward the cure of this troublesome affection is the formation of a habit whereby one shall have a certain time for defecating. One action of the bowels should be desired every day, and this enemata may be resorted to each day till such an habit is acquired. If possible, however, the bowels should be kept open by dietetic and hygienic measures. A diet composed of coarse food will usually stimulate the bowels to action, and is not ordinarily followed by that sluggish condition so often observed after the administration of laxative or purgative drugs.

The habitual use of purgative medicines is to be deprecated. Sometimes they are required and are then conducive of much good. An ideal aperient would so act as to cause the bowels to move and at the same time tone up the lethargic muscular tunic of the gut, and prevent its relapsing into its former state of inactivity. Such a remedy is not to be found in the materia medica. A pill composed of a quarter grain each of ex. nucis. vom., ex. belladonna and podophyl., and two grains of aloes, has worked best in your scribe's limited experience. The crude drugs act better than the alkaloids, for the simple reason that they possess properties not contained in the derivatives. The olfactory nerves, when subjected to effluviums for a considerable time, cease to perceive them, and likewise the nerves of the large intestine when subjected to stimulants or irritants for any lengthy period become blunted. Larger doses are continually being required to produce an evacuation, and the irritation caused by these large doses will, in the end, cause an inflammation of the mucous membrane of the bowel.

In conclusion, I will state that in my opinion medicine is only serviceable in chronic constipation as an aid to the more important measures mentioned previously in this paper. A stated time to defecate, a suitable place to perform the act, together with the enema and diet, will suffice to cure most cases of chronic constipation when not due to mechanical causes.

